# Exercise Attenuates Doxorubicin-Induced Myocardial Injury by Inhibiting TSHR and Regulating Macrophage Polarization Through miR-30d-5p/GALNT7

**DOI:** 10.1155/2024/5562293

**Published:** 2024-10-26

**Authors:** Haiyan Wu, Ruoyu Zhou, Hanxin Kong, Jieqiong Yang, Suijuan Liu, Xiaolin Wei, Kunzhi Li, Yunmei Zhang

**Affiliations:** ^1^Faculty of Life Science and Technology, Kunming University of Science and Technology, Kunming 650500, Yunnan, China; ^2^Department of Cardiology, The First People's Hospital of Yunnan Province, The Affiliated Hospital of Kunming University of Science and Technology, Kunming 650032, Yunnan, China; ^3^Medical School, Kunming University of Science and Technology, Kunming 650500, Yunnan, China

**Keywords:** doxorubicin, exercise, macrophage polarization, miR-30d-3p, myocardial injury

## Abstract

**Objective:** Doxorubicin (DOX) is an extensively used chemotherapeutic agent that induces cardiotoxicity. Studies have reported that exercise (EXE) can alleviate DOX-induced cardiotoxicity. Therefore, this study aimed to explore the mechanism by which EXE attenuates DOX-induced myocardial injury.

**Methods:** In this study, cell and animal models of DOX-induced myocardial injury were constructed. The animal model was subjected to EXE intervention.

**Results:** In this study, in vitro experiments revealed that miR-30d-5p negatively regulated polypeptide N-acetylgalactosaminyltransferase 7 (GALNT7) and that GALNT7 negatively regulated the expression of thyroid stimulating hormone receptor (TSHR). miR-30d-5p downregulated the expression of GALNT7, promoted the expression of TSHR, and promoted macrophage M1 polarization, thus aggravating cardiomyocyte injury. In vivo experiments revealed that EXE intervention significantly downregulated miR-30d-5p and TSHR expression, upregulated GALNT7, reduced inflammation, and promoted M2 macrophage polarization, thereby alleviating DOX-induced myocardial injury. In addition, overexpression of miR-30d-5p or knockdown of GALNT7 weakened the intervention effect of EXE, whereas overexpression of GALNT7 or knockdown of TSHR promoted the effect of EXE.

**Conclusion:** EXE can modulate the miR-30d-5p/GALNT7 axis to inhibit the expression of TSHR, thereby regulating the polarization of macrophages to the M2 phenotype and ultimately alleviating DOX-induced myocardial injury, which provides new targets and strategies for the clinical treatment of myocardial injury.

## 1. Introduction

Doxorubicin (DOX) belongs to the anthracycline family of anticancer drugs. It is a common and effective chemotherapeutic agent utilized to treat different diseases, including lymphoma, leukemia, breast cancer, and solid tumors [1]. However, DOX lacks tumor specificity and has serious side effects, such as nephrotoxicity, muscular toxicity, myelosuppression, induction of testicular oxidative stress, and cardiotoxicity [2–4]. DOX leads to significant deterioration of mitochondrial function and dynamic regulation; increased cardiomyocyte oxidative stress, inflammation, myocardial injury, and apoptosis; and ultimately impaired cardiac function [5]. Moreover, as a widely used chemotherapeutic agent, DOX induces cardiac toxicity, which reduces the quality of life of patients and leads to cardiac arrhythmia, myocardial ischemia, and life-threatening cardiomyopathy [6]. Therefore, developing efficient and safe adjuvant interventions to alleviate DOX-induced myocardial injury and improve the safety of its clinical application is highly important.

Numerous studies have shown that exercise (EXE) intervention has beneficial effects on DOX-induced myocardial injury. For example, an EXE intervention was shown to reduce acute and late DOX-induced cardiotoxicity [7]. Another study found that endurance EXE can reduce cardiomyocyte apoptosis and alleviate DOX-induced cardiotoxicity [8]. Furthermore, EXE can induce defenses by increasing the expression of mitochondria-specific adenosine triphosphate (ATP)-binding cassette transporters and reducing mitochondrial DOX accumulation [9]. However, there are few reports on the molecular mechanism by which EXE attenuates DOX-induced myocardial injury.

MicroRNAs (miRNAs) are a class of endogenous noncoding ribonucleic acids (RNAs) that regulate gene expression and are involved in various physiological and pathological processes [10]. miR-30d-5p is a type of miRNA that plays a significant role in regulating cell proliferation, motility, apoptosis, autophagy, tumorigenesis, and chemical resistance. Its abnormal levels are associated with a variety of diseases [11]. It was shown to be upregulated in hypertrophic cardiomyopathy [12]. Another study found that resveratrol protects H9c2 cardiomyocytes from hypoxia-induced apoptosis via the miR-30d-5p/SIRT1/NF-*κ*B axis [13]. miR-30d-5p derived from neutrophils was found to induce polarization in M1 macrophages [14]. In addition, EXE was reported to cause changes in miRNA expression, which in turn affected the progression of diabetic heart disease [15]. Moreover, miR-30d-5p has been found to alter the effect of EXE on myocardial injury [16]. Whether EXE can regulate the expression of miR-30d-5p, affect the polarization of macrophages, and alleviate DOX-induced myocardial damage is still unclear; therefore, this study explored the regulatory mechanisms affected by EXE.

Polypeptide N-acetylgalactosaminyltransferase 7 (GALNT7) is a glycosyltransferase that plays an important role in disease progression [17]. GALNT7 can affect T-cell proliferation and participate in the immune response [18]. Recent studies have shown that GALNT7 is positively correlated with the abundance of various immune cell types [19]. In addition, during thyroid cancer progression, miR-30d-5p inhibits thyroid cancer progression by targeting GALNT7, and GALNT7 has been demonstrated to be a direct and functional target of miR-30b-5p [20]. However, whether GALNT7 can regulate macrophage polarization and if it has a role in DOX-induced myocardial injury have not been reported. This study further explored the mechanism of action of the miR-30d-5p/GALNT7 axis in DOX-induced myocardial injury.

The thyroid stimulating hormone (TSH) receptor (TSHR) is widely expressed in a variety of tissues and cells and is involved in thyroid diseases and neuroendocrine immunomodulatory networks [21]. TSH can induce cardiac dysfunction [22]. In myocardial tissue, the expression of functional TSHR has been shown to influence cardiac electrical properties [23]. In addition, TSHR can be expressed in macrophages, has proinflammatory effects on macrophages, and promotes vascular inflammation and atherosclerosis [24]. However, the mechanism of action of TSHR in DOX-induced myocardial injury remains unclear.

On the basis of the above research background, we speculate that the molecular axis of miR-30d-5p/GALNT7/TSHR may affect the polarization of macrophages. However, there are no reports that EXE regulates macrophage polarization through miR-30d-5p/GALNT7/TSHR to attenuate DOX-induced myocardial injury. Therefore, this study explored the molecular mechanism by which EXE, through miR-30d-5p/GALNT7, inhibits TSHR regulation of macrophage polarization to alleviate DOX-induced myocardial injury, hoping to provide new ideas for the treatment of DOX-induced myocardial injury and improve the safety of its clinical application.

## 2. Materials and Methods

### 2.1. Cell Culture

H9C2 cells and human mononuclear (THP-1) cells were purchased from Otwo Biotechnology Co., Ltd. (Shenzhen, China). H9C2 cells were cultured in Dulbecco's modified Eagle medium (DMEM) (Gibco, United States) supplemented with 10% FBS, 100 U/mL penicillin, and 100 mg/mL streptomycin. THP-1 cells were cultured in RPMI 1640 medium (Gibco). THP-1 macrophages were generated by treating THP-1 cells with 100 nM phorbol 12-myristate 13-acetate (PMA; Sigma‒Aldrich) for 24 h and then culturing them for 3 days without PMA, and M0 cells with a nonpolarized phenotype were obtained. To produce a polarized phenotype, THP-1 macrophages were treated with 20 ng/mL interleukin-4 (IL-4) [25] for 72 h to induce M2 macrophage polarization. Both cell lines were grown in air containing 5% CO_2_ at 37°C.

### 2.2. Cell Transfection

The miR-30d-5p mimic and the negative control (NC) mimic were designed by Hunan Fenghui Biotechnology Co., Ltd. GALNT7 and TSHR overexpression plasmids and corresponding NC groups were designed and constructed. When the THP-1-derived macrophages density reached approximately 80%–90%, the miRNA mimics and plasmids were transfected into the THP-1-derived macrophages with lipofectamine 3000 transfection reagent (Invitrogen, United States) according to the manufacturer's protocol, and the medium was changed 8 h after transfection, followed by the subsequent experiments 48 h later.

### 2.3. Generation of the Myocardial Injury Cell Model Induced by DOX

H9C2 cardiomyocytes were seeded into six-well culture plates at a density of 5 × 10^4^ cells/mL and then treated with 5 µM [26] DOX (Sigma–Aldrich, United States) for 24 h to establish a cell model of DOX-induced myocardial injury.

### 2.4. Construction of an Animal Model of DOX-Induced Myocardial Injury

Male 5–6-week-old Sprague Dawley (SD) rats weighing 150–170 g were purchased from the Experimental Animal Center of Kunming Medical University. The animal study was reviewed and approved by the Experimental Animal Center Committee of Kunming University of Science and Technology. The rats were allowed to adapt to the environment for 1 week at 22–25°C on a 12-h diurnal cycle. One week later, the rats were randomly divided into seven groups (*n* = 10): the control group (without EXE intervention), DOX group, DOX + EXE group, DOX + EXE + miR-30d-5p mimic group, DOX + EXE + miR-30d-5p mimic + oe-GALNT7 group, DOX + EXE + si-GALNT7 group, and DOX + EXE + si-GALNT7 + si-TSHR group. The rat model was established by intraperitoneal injection of DOX at a dose of 2 mg/kg once a week for 6 weeks following the protocol used in previous studies [27, 28], including our laboratory studies. In addition, the miR-30d-5p mimic, GALNT7 overexpression plasmid, and GALNT7 and TSHR siRNAs (GenePharma, China) were injected through the tail vein during the EXE cycle.

### 2.5. EXE Program

**Figure 1:**
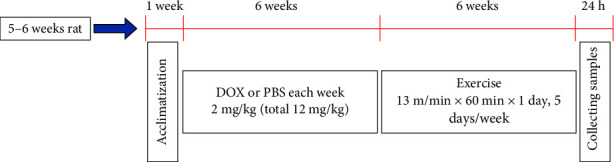
Animal protocol. DOX, doxorubicin.

The EXE program followed previously published procedures [8]. In this study, a 6-week EXE intervention was chosen. As shown in Figure 1, the rats were allowed to adapt for 1 week, and then the animal model was established by injecting DOX for 6 weeks. Two days after the last injection of DOX, the rats assigned to the EXE intervention exercised on an animal treadmill at a speed of 13 m/min for 60 min/day, 5 days a week, for 6 weeks.

### 2.6. Assessment of Cardiac Function

Cardiac function was assessed by echocardiography. All echocardiographic data were stored and analyzed in accordance with the current standardized measurements and analyses in China. The rats were anesthetized via inhalation of 1.5% isoflurane and kept warm on a heated platform. The anterior chest was shaved, and the rats were placed in the left lateral decubitus position. Images of the left ventricle and cardiac function parameters were acquired with a GE Vivid E95 ultrasound machine (GE Healthcare, Horten, Norway) equipped with an L8-18iD transducer with a frequency of 5–18 MHz. M-model echocardiography guided with two-dimensional images was used to assess the left ventricular systolic function with a parasternal long-axis view. The left ventricular ejection fraction (LVEF) and left ventricular fraction shortening (LVFS) were calculated throughout eight consecutive cardiac cycles. Higher LVEF and LVFS values indicate better cardiac function. The echocardiogram data were analyzed by researchers who were blinded to the treatment.

### 2.7. Animal Body and Heart Weights

The initial and final body weights of all test animals were measured at the beginning and end of the trial. The heart tissue was collected and weighed after the study, an accurate body weight was measured using a sensitive weighing scale, and differences between the test groups were recorded.

### 2.8. Measurement of the Myocardial Injury Index

According to the kit instructions, cardiac troponin T (cTnT; SEKR-0047, Solarbio, Beijing, China), lactate dehydrogenase (LDH; BC0685, Solarbio, Beijing, China), and aspartate aminotransferase (AST; ml092714, Enzyme-Linked Biology, Shanghai, China) were detected in the rat serum. In brief, the serum of each rat was collected and added to the reaction mixture. The absorbance value was measured with a microplate reader and the contents of cTnT, LDH, and AST were calculated according to the corresponding multipoint calibration curves. If the absorption value of a sample was not within the range of the measurement calibration curve, it was diluted or the sample size was increased for retesting.

### 2.9. Hematoxylin and Eosin (H&E) Staining

Paraffin sections of rat heart tissues were dewaxed, hydrated with graded ethanol, stained with hematoxylin water, differentiated with acid and ammonia water, rinsed with running water, stained with alcohol and eosin, and dried at room temperature; xylene was applied for transparency and resin was used for sealing. The results were observed under a microscope and photographed (400857, Nikon, Japan).

### 2.10. TUNEL

Briefly, myocardial tissue sections and cells were fixed on ice with fixation solution for 15 min. Phosphate buffered saline (PBS) was used to wash and permeabilize the samples on ice with 0.1% Triton X-100 and 0.1% sodium citrate for 2 min. The samples were then washed with PBS three times and incubated with the TUNEL reaction mixture for 1 h. Finally, apoptotic cells were identified via fluorescence microscopy (Nikon, Japan). Red fluorescence represents apoptotic cells, and the experiment was repeated at least three times.

### 2.11. Western Blot

Total protein was extracted from the tissues and cells via radioimmunoprecipitation assay (RIPA) buffer (Beyotime, Shanghai, China), and the protein concentration was determined with a bicinchoninic acid (BCA) protein analysis kit (Beyotime, Shanghai, China). The proteins were subsequently separated by electrophoresis on a 10% sodium dodecyl sulfate (SDS)‒polyacrylamide gel. After the proteins were transferred to a polyvinylidene fluoride (PVDF) membrane and blocked with 5% skim milk powder for 1 h at room temperature, the membrane was incubated with primary antibodies against the following: inducible nitric oxide synthase (iNOS,1 : 1000, ab178945, Abcam, United Kingdom), monocyte chemoattractant protein-1 (MCP-1,1 : 1000, ab7202, Abcam, United Kingdom), Ym1 (1 : 10,000, ab192029, Abcam, United Kingdom), transforming growth factor-beta 1 (TGF-*β*1,1 : 1000, ab215715, Abcam, United Kingdom), interleukin-10 (IL-10,1 : 1000, ab290735, Abcam, United Kingdom), Arg1 (1 : 1000, ab124917, Abcam, United Kingdom), Bcl-2 (1 : 2000, ab182858, Abcam, United Kingdom), Bax (1 : 1000, ab32503, Abcam, United Kingdom), cleaved caspase 3 (1 : 5000, ab214430, Abcam, United Kingdom), GALNT7 (1 : 5000, ab97645, Abcam, United Kingdom), and glyceraldehyde 3-phosphate dehydrogenase (GAPDH, 1 : 1000, ab9485, Abcam, United Kingdom) at 4°C overnight. After being washed with Tris Buffered Saline and Tween 20 (TBST), the membranes were incubated with the corresponding secondary antibody for 1 h. The signal was developed via an enhanced chemiluminescence kit (Millipore, Burlington, MA, United States). Semiquantitative analysis of the protein bands was performed using Image J software, and GAPDH was used as an internal reference protein.

### 2.12. Immunohistochemistry

The myocardial tissue was fixed and embedded in paraffin. Tissue sections of 5 μm thickness were then dewaxed, rehydrated, and treated with 0.3% H_2_O_2_. After rinsing, the sections were blocked with 5% normal goat serum, and antigen retrieval was performed. The sections were then incubated overnight at 4°C with antibodies against CD11b (1:200, ab133357) and CD206 (1:200, ab64693). Next, the sections were washed three times with PBS and incubated with the corresponding secondary antibodies. The results were observed and photographed under a fluorescence microscope (Nikon, Japan).

### 2.13. Determination of Inflammatory Factors

Rat IL-1*β* (SEKR-0002), rat IL-6 (SEKR-0005), and rat TNF-*α* (SEKR-0009) kits were purchased from Solarbio, Beijing, China. The cell culture supernatant or tissue homogenate was subjected to enzyme-linked immunosorbent assay (ELISA) according to the manufacturer's instructions. The supernatant was extracted, and the reaction reagents were added. After the samples were allowed to react, the contents of IL-1*β*, IL-6, and TNF-*α* in the tissues and cells were detected with a microplate reader at 450 nm.

### 2.14. Reverse Transcription-Quantitative Polymerase Chain Reaction (RT-qPCR)

A TRIzol RNA extraction kit (Invitrogen, Carlsbad, CA, United States) was used to extract total RNA from the collected cells and tissues. The extraction steps were carried out in strict accordance with instructions of the kit. The first strand of complementary deoxyribonucleic acid (cDNA) (Takara, Japan) was subsequently assembled using the total RNA of the sample as a template, and the cDNA obtained was used as a template for qPCR amplification. The qPCR analyses were completed with a PCR assay system and SYBR Premix Ex Taq (Vazyme). U6 or GAPDH was used as an internal reference gene to normalize the expression of the other genes. The expression levels of the genes were calculated using the 2^−*ΔΔ*Ct^ method. The primer sequences are displayed in Table 1.

### 2.15. Detection of Macrophage Markers by Flow Cytometry

The cells from each group were collected and cultured on ice for 30 min with one of the following antibodies: anti-CD11b (ab184308, Abcam), anti-CD86 (ab239075, Abcam), anti-CD206 (ab270682, Abcam), or anti-CD163 (ab182422, Abcam). The fluorescence was detected via flow cytometry, and the data were analyzed using FlowJo 10.8.1.

### 2.16. Dual-Luciferase Reporter Assay

The GALNT7 3′ untranslated regions (3′UTRs) wild-type (WT) or mutant (MUT) sequence containing the miR-30d-5p binding site was inserted into a pmirGLO luciferase target expression vector (PuFei Biology, China), and the pmirGLO-GALNT7-WT/MUT luciferase reporter vector was constructed. Then, the vectors were cotransfected with the miR-30d-5p mimic or NC mimic into 293T cells. After transfection for 48 h, luciferase activity was detected.

### 2.17. Coimmunoprecipitation (Co-IP)

THP-1 cells were collected and lysed with cell lysis buffer. The cell lysates were incubated with anti-GALNT7 or TSHR antibodies or normal immunoglobulin G (IgG) overnight at 4°C, followed by incubation with protein A and protein G (A/G) agarose (Santa Cruz Biotechnology, Santa Cruz, CA, United States) for 4 h at 4°C. Finally, the protein complexes were washed and subjected to Western blot detection. Whole-cell lysates were used as input controls, and normal IgG was used as a NC.

### 2.18. Cell Proliferation

H9C2 cells (1 × 10^4^) were inoculated into 96-well plates and cultured for 24 h. After adding cell counting kit-8 (CCK-8) solution to the sample and modulating the microplate reader, the absorbance was measured at a wavelength of 450 nm (ELX800, BioTeK, United Kingdom). According to the absorbance values obtained in the experimental group and the control group, the cell viability was calculated.

### 2.19. Data Statistics

Statistical analysis was performed using GraphPad Prism 8.0 software. The data are expressed as mean ± standard deviation (SD), and the differences between groups were tested by one- or two-way analysis of variance (ANOVA) and Student's *t* test.

## 3. Results

### 3.1. EXE Inhibits DOX-Induced Myocardial Inflammation and Apoptosis in Rats by Regulating Macrophage Polarization

**Figure 2:**
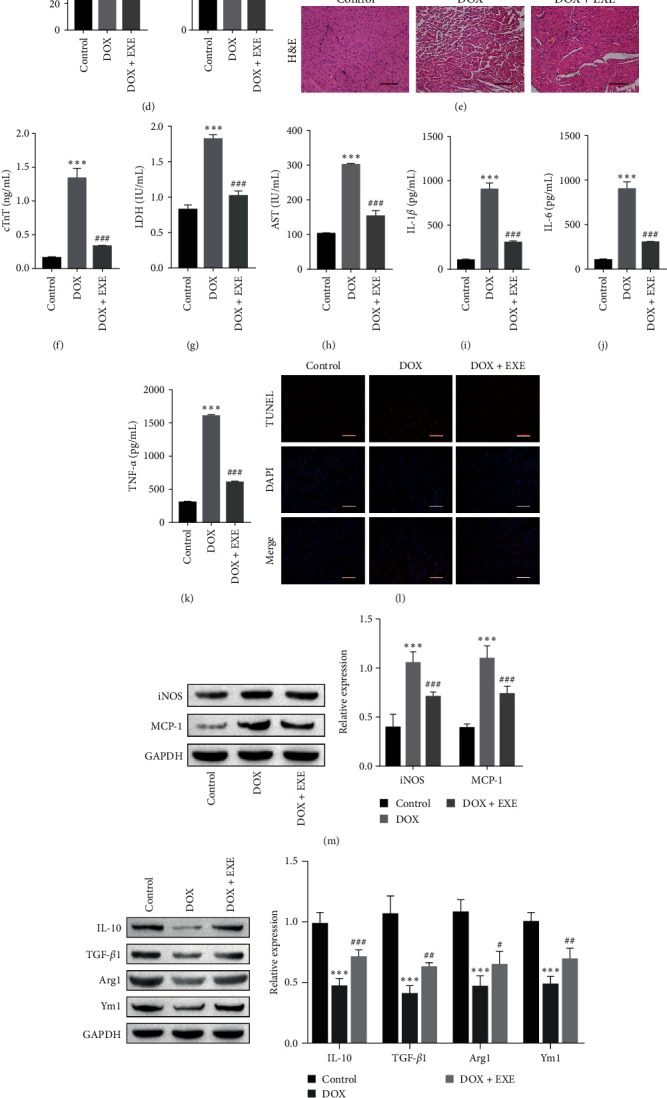
Exercise inhibits DOX-induced myocardial inflammation and apoptosis in rats. (A, B) The body weight and heart weight of DOX-induced rats were detected after EXE intervention. (C, D) Echocardiographic results showed that EXE intervention significantly improved LVEF and LVFS in DOX-induced rats, indicating improved cardiac function due to EXE intervention. (E) Pathological changes of myocardial tissue observed by H&E staining, scale bar = 100 μm. (F–H) EXE intervention decreased the myocardial injury indices in serum of rats (cTnT, LDH, and AST). (I–K) ELISA was used to measure the levels of inflammatory cytokines IL-1*β*, IL-6, and TNF-*α* in rat myocardial tissue, and EXE intervention inhibited the expression of inflammatory factors. (L) TUNEL results showed that EXE intervention inhibited apoptosis of rat cardiomyocytes, scale bar = 100 μm; (M, N) The expression of macrophage markers (iNOS, MCP-1, IL-10, TGF-*β*1, Arg1, and Ym1) was detected by Western blot. (O) The expression of macrophage markers CD11b and CD206 was detected in rat myocardial tissue by immunohistochemistry, scale bar = 100 μm. (P) RT-qPCR results showed that the expression of miR-30d-5p in rat myocardial tissue was decreased after EXE intervention. (Q) GALNT7 expression was detected by RT-qPCR, and EXE intervention promoted GALNT7 expression. (R) The results of RT-qPCR showed that EXE intervention inhibited the expression of TSHR. One-way ANOVA was used for statistical analysis. The data are expressed as mean ± SD. *⁣*^*∗∗*^*p* < 0.01, *⁣*^*∗∗∗*^*p* < 0.001 vs. control; ^#^*p* < 0.05, ^##^*p* < 0.01, ^###^*p* < 0.001 vs. DOX. Control: normal rats without exercise intervention. AST, aspartate aminotransferase; cTnT, cardiac troponin T; DOX, doxorubicin; EXE, exercise; LDH, lactate dehydrogenase; LVEF, left ventricular ejection fraction; LVFS, left ventricular fraction shortening.

The animal body weight and heart weight were severely reduced in the DOX-treated group, whereas the EXE intervention group, after DOX treatment, presented remarkable increases in body weight and heart weight (Figure 2A,B). Compared with those in the control group, the echocardiographic findings revealed that the left ventricles were enlarged, the wall motion was attenuated, and the LVEF and LVFS were reduced after DOX treatment. Compared with those in the DOX group, LVEF and LVFS were significantly improved in the DOX + EXE group, indicating improved cardiac function due to EXE intervention (Figure 2C,D). H&E staining revealed that the myocardial tissue of the control group presented a typical structure of myocardial cells, and the myocardial cells were arranged in order. Compared with those of the control group, the myocardial tissue of the DOX-treated group presented abnormal morphology, including extensive cytoplasmic vacuolation, myofibrillar loss, inflammatory cell infiltration, and tissue structure dispersion. However, EXE intervention after DOX treatment strongly attenuated myocardial injury (Figure 2E).

The levels of cTnT, LDH, and AST are commonly used biomarkers for the diagnosis of myocardial injury, and elevated levels of cTnT, LDH, and AST indicate cardiac injury. In the rat serum, the levels of cTnT, LDH, and AST were markedly greater in the DOX treatment group than in the control group and were decreased by EXE intervention (Figure 2F–H). DOX-induced a significant increase in the contents of IL-1*β*, IL-6, and TNF-*α*, which were markedly reduced by EXE intervention after DOX treatment (Figure 2I–K). TUNEL results revealed that DOX-induced cardiomyocyte apoptosis, which was reduced after EXE intervention (Figure 2L). Macrophage infiltration is the main cause of myocardial injury [29]. The Western blot data indicated that the expression of M1 macrophage markers (iNOS and MCP-1) was increased and that the expression of M2 macrophage markers (IL-10, TGF-*β*1, Arg1, and Ym1) was decreased after DOX treatment. These effects were reversed by EXE intervention after DOX treatment, suggesting that EXE regulates macrophage M2 polarization to alleviate DOX-induced myocardial injury (Figure 2M,N). The immunohistochemical results led to similar conclusions as previously described (Figure 2O). The RT-qPCR data revealed that miR-30d-5p and TSHR were upregulated and that GALNT7 was downregulated in the DOX-treated group, whereas EXE intervention after DOX treatment markedly decreased the levels of miR-30d-5p and TSHR and increased the content of GALNT7 (Figure 2P‒R). In summary, DOX aggravated inflammation, promoted myocardial injury and apoptosis, induced M1 polarization in macrophages, upregulated the expression of miR-30d-5p and TSHR, and downregulated the expression of GALNT7, whereas EXE intervention significantly alleviated DOX-induced myocardial injury.

### 3.2. miR-30d-5p Inhibits M2 Macrophage Polarization by Regulating GALNT7

**Figure 3:**
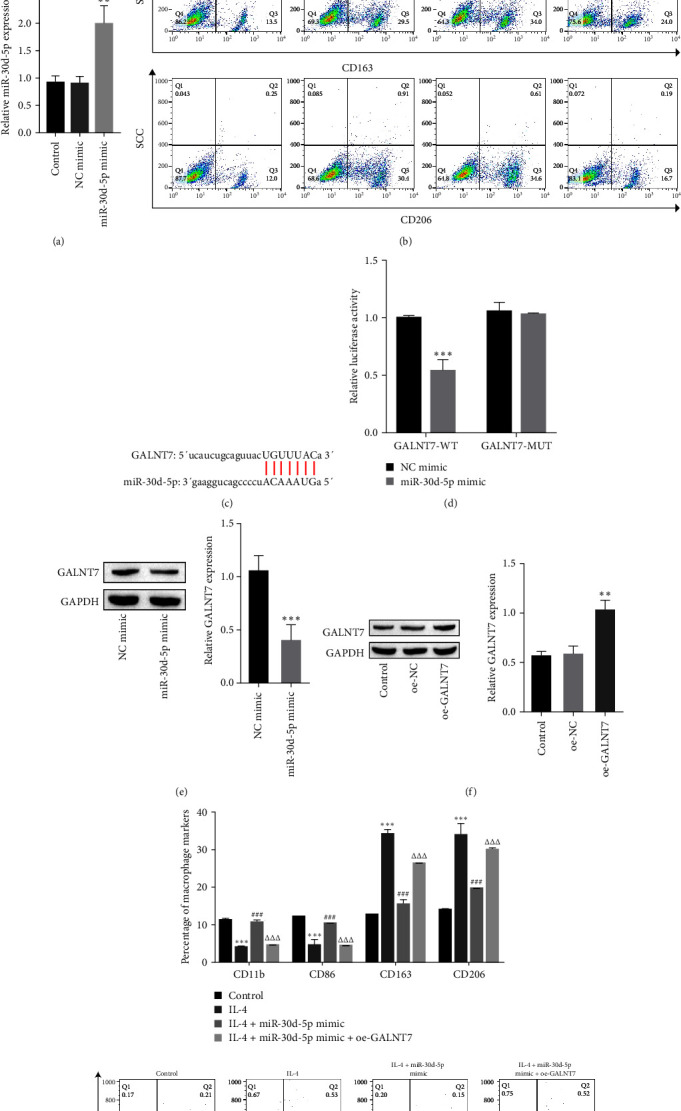
miR-30d-5p affects macrophage polarization by regulating GALNT7. (A) RT-qPCR assays revealed that miR-30d-5p levels were increased after transfection with the miR-30d-5p mimic into THP-1-derived macrophages; one-way ANOVA was used for statistical analysis. (B) The expression of the macrophage polarization markers CD11b, CD86, CD206, and CD163 was detected by flow cytometry, and the miR-30d-5p mimic inhibited macrophage M2 polarization. Two-way ANOVA was used for statistical analysis. (C) The binding sequence of miR-30d-5p and GALNT7. (D) The targeting of miR-30d-5p to GALNT7 was verified by a dual-luciferase reporter assay, and two-way ANOVA was used for statistical analysis. *⁣*^*∗∗∗*^*p* < 0.001 vs. NC mimic. (E) Western blot analysis revealed that the miR-30d-5p mimic inhibited the expression of GALNT7, and Student's *t* test was used for statistical analysis; *⁣*^*∗∗∗*^*p* < 0.001vs. the NC mimic. (F) Western blotting was used to detect the overexpression efficiency of GALNT7; one-way ANOVA was used for statistical analysis. (G) After the cells were transfected with the miR-30d-5p mimic or oe-GALNT7, the expression of CD11b, CD86, CD163, and CD206 in the macrophages was detected by flow cytometry, and two-way ANOVA was used for statistical analysis. The data are expressed as mean ± SD. *⁣*^*∗∗*^*p* < 0.01, *⁣*^*∗∗∗*^*p* < 0.001 vs. the control; ^###^*p* < 0.001 vs. the IL-4; ^ΔΔΔ^*p* < 0.001 vs. the IL-4 + miR-30d-5p mimic. Control: normal macrophages without any treatment. NC, negative control; SD, standard deviation.

To explore whether miR-30d-5p can inhibit macrophage M2 polarization, markers of macrophage polarization were detected after the overexpression of miR-30d-5p. miR-30d-5p mimic was transfected into THP-1-derived macrophages. The transfection efficiency of the miR-30d-5p mimic is shown in Figure 3A. The expression of miR-30d-5p increased after transfection with the miR-30d-5p mimic, indicating successful transfection. The flow cytometry data revealed that the expression levels of M1 macrophage markers (CD11b and CD86) were markedly decreased, whereas the expression levels of M2 macrophage markers (CD206 and CD163) were strongly increased in the IL-4 group (positive control group in which macrophages were induced toward M2 polarization) and the IL-4 + NC mimic group. Compared with the IL-4 + NC mimic, the miR-30d-5p mimic reversed the expression of M2 macrophage markers induced by IL-4, indicating that miR-30d-5p inhibited the polarization of macrophages to the M2 phenotype (Figure 3B).

To confirm that miR-30d-5p inhibits macrophage M2 polarization by regulating GALNT7, the StarBase (https://starbase.sysu.edu.cn/) database was used to predict that GALNT7 is a potential target gene of miR-30d-5p. The binding sequence is shown in Figure 3C. Furthermore, the miR-30d-5p mimic markedly inhibited the fluorescence intensity of the GALNT7 WT plasmid, whereas the fluorescence intensity of the MUT plasmid did not differ substantially from that of the control group (Figure 3D). The Western blot results indicated that the level of GALNT7 decreased after the cells were transfected with the miR-30d-5p mimic (Figure 3E). These findings revealed that miR-30d-5p targets negatively regulate GALNT7 expression.

To further determine the function of miR-30d-5p in regulating GALNT7 in macrophage polarization, we overexpressed GALNT7 to detect the expression of macrophage markers. The overexpression efficiency of GALNT7 is shown in Figure 3F. Further detection by flow cytometry revealed that IL-4 induced macrophages to polarize to the M2 phenotype and that the overexpression of miR-30d-5p attenuated the induction of IL-4, whereas the simultaneous overexpression of miR-30d-5p and GALNT7 restored the induction of IL-4 (Figure 3G). In conclusion, miR-30d-5p negatively regulates GALNT7 expression and inhibits macrophage polarization to the M2 phenotype.

### 3.3. miR-30d-5p/GALNT7 Regulates M1 Macrophage Polarization by Promoting TSHR

**Figure 4:**
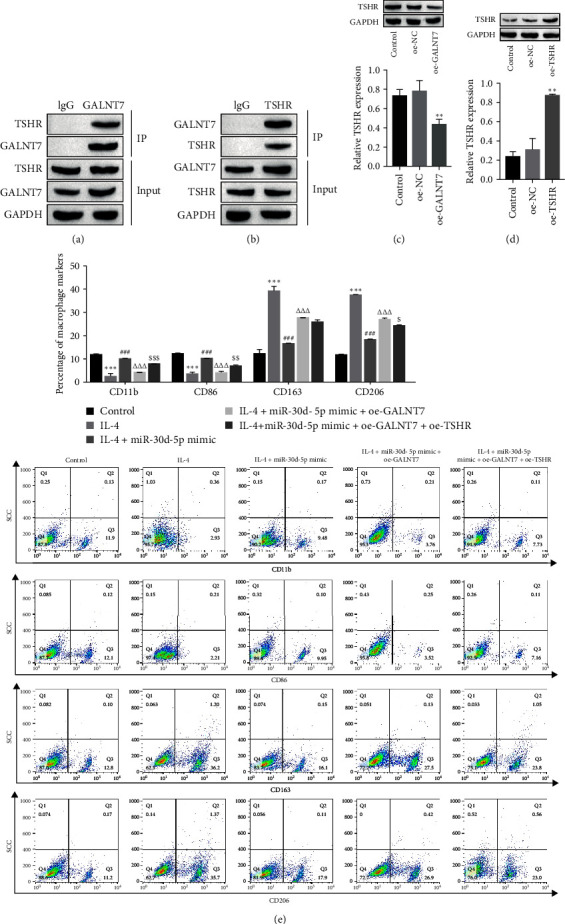
miR-30d-5p/GALNT7 regulates macrophage polarization by inhibiting TSHR. (A, B) The interaction between GALNT7 and TSHR was determined by co-IP. (C) Western blot analysis revealed that the expression of TSHR decreased after transfection with oe-GALNT7 into THP-1-derived macrophages. (D) The overexpression efficiency of TSHR was detected by Western blotting. (E) After transfection with the miR-30d-5p mimic oe-GALNT7 or oe-TSHR, the expression of CD11b, CD86, CD163, and CD206 in macrophages was detected by flow cytometry. One- or two-way ANOVA was used for statistical analysis. The data are expressed as the means ± SD. *⁣*^*∗∗*^*p* < 0.01, *⁣*^*∗∗∗*^*p* < 0.001 vs. control; ^###^*p* < 0.001 vs. IL-4; ^ΔΔΔ^*p* < 0.001 vs. IL-4 + miR-30d-5p mimic; ^$^*p* < 0.05, ^$$^*p* < 0.01, ^$$$^*p* < 0.001 vs. IL-4 + miR-30d-5p mimic+oe-GALNT7. Control: normal macrophages without any treatment. co-IP, coimmunoprecipitation; SD, standard deviation; TSHR, thyroid stimulating hormone receptor.

To determine the interaction between GALNT7 and TSHR, we performed co-IP experiments and showed that TSHR was enriched in GALNT7 immuno-pretreatment products (Figure 4A,B). Furthermore, GALNT7 and TSHR overexpression plasmids were transfected into the THP-1-derived macrophages. The level of TSHR was reduced after overexpression of GALNT7 (Figure 4C), suggesting that GALNT7 negatively regulates the expression of TSHR. To confirm that GALNT7 regulates macrophage polarization through TSHR, we overexpressed TSHR, and the overexpression efficiency is shown in Figure 4D. Flow cytometry revealed that macrophages in the IL-4 group were polarized to the M2 phenotype, macrophages in the IL-4 + miR-30d-5p mimic group were polarized to the M1 phenotype, and macrophages in the IL-4 + miR-30d-5p mimic+oe-GALNT7 group were polarized to the M2 phenotype. However, simultaneous overexpression of miR-30d-5p, GALNT7, and TSHR increased macrophage polarization toward the M1 phenotype (Figure 4E). These results suggest that miR-30d-5p regulates M1 macrophage polarization by promoting TSHR expression through GALNT7.

### 3.4. The miR-30d-5p/GALNT7/TSHR Molecular Axis Promotes Cardiomyocyte Injury by Regulating Macrophage Polarization

**Figure 5:**
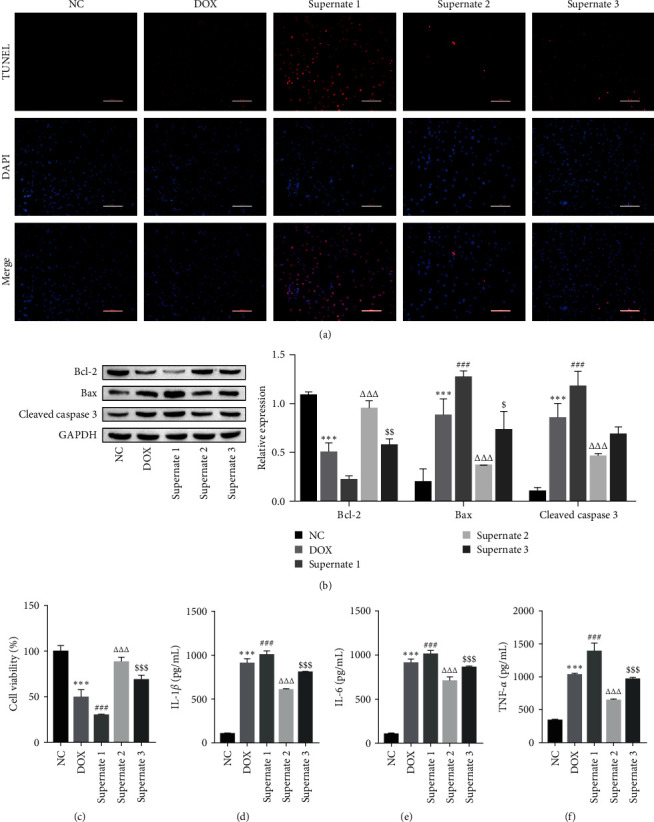
The miR-30d-5p/GALNT7/TSHR molecular axis promotes cardiomyocyte injury by regulating macrophage polarization. (A) After coculture with the supernatant of THP-1 cells transfected with the miR-30d-5p mimic oe-GALNT7 or oe-TSHR, the level of cardiomyocyte apoptosis was detected by TUNEL, scale bar = 100 μm. (B) The expression of the apoptosis-related proteins Bcl-2, Bax, and cleaved caspase 3 was detected by Western blot. (C) Cardiomyocyte proliferation was assessed by the CCK-8 assay. (D‒F) The levels of inflammatory factors in H9C2 cells were measured by ELISA. One- or two-way ANOVA was used for statistical analysis. The data are expressed as mean ± SD. *⁣*^*∗∗∗*^*p*  < 0.001 vs. NC; ^##^*p*  < 0.01, ^###^*p*  < 0.001 vs. DOX; ^ΔΔΔ^*p*  < 0.001 vs. Supernate 1; ^$^*p*  < 0.05, ^$$^*p*  < 0.01, ^$$$^*p*  < 0.001 vs. Supernate 2. DOX, doxorubicin; NC, negative control of H9C2 cells; SD, standard deviation; TSHR, thyroid stimulating hormone receptor.

To assess the function of the miR-30d-5p/GALNT7/TSHR molecular axis in alleviating cardiomyocyte injury by regulating macrophage polarization, the experimental samples were divided into the NC group (NC of H9C2 cells), the DOX group (model group), the supernatant 1 group (the supernatant of THP-1-derived macrophages transfected with the miR-30d-5p mimic was cocultured with myocardial injury cells), the supernatant 2 group (the supernatant of THP-1-derived macrophages transfected with the miR-30d-5p mimic + oe-GALNT7 was cocultured with myocardial injury cells), and the supernatant 3 group (the supernatant of THP-1-derived macrophages transfected with the miR-30d-5p mimic+oe-GALNT7+oe-TSHR was cocultured with myocardial injury cells). TUNEL, Western blot, CCK-8, and ELISA results revealed that after cardiomyocytes were incubated with supernatant 3, the molecular axis of miR-30d-5p/GALNT7/TSHR promoted the DOX-induced apoptosis of H9C2 cells by regulating the M1 polarization of macrophages (Figure 5A), increasing the expression of Bax and cleaved caspase 3, reducing the expression of Bcl-2 (Figure 5B), inhibiting DOX-induced H9C2 cell proliferation (Figure 5C), and increasing the levels of IL-1*β*, IL-6, and TNF-*α* in H9C2 cells (Figure 5D‒F). These findings indicate that the miR-30d-5p/GALNT7/TSHR molecular axis promotes cardiomyocyte injury by regulating M1 macrophage polarization, promoting apoptosis, and inhibiting cell proliferation.

### 3.5. EXE Alleviates DOX-Induced Myocardial Injury Through miR-30d-5p

**Figure 6:**
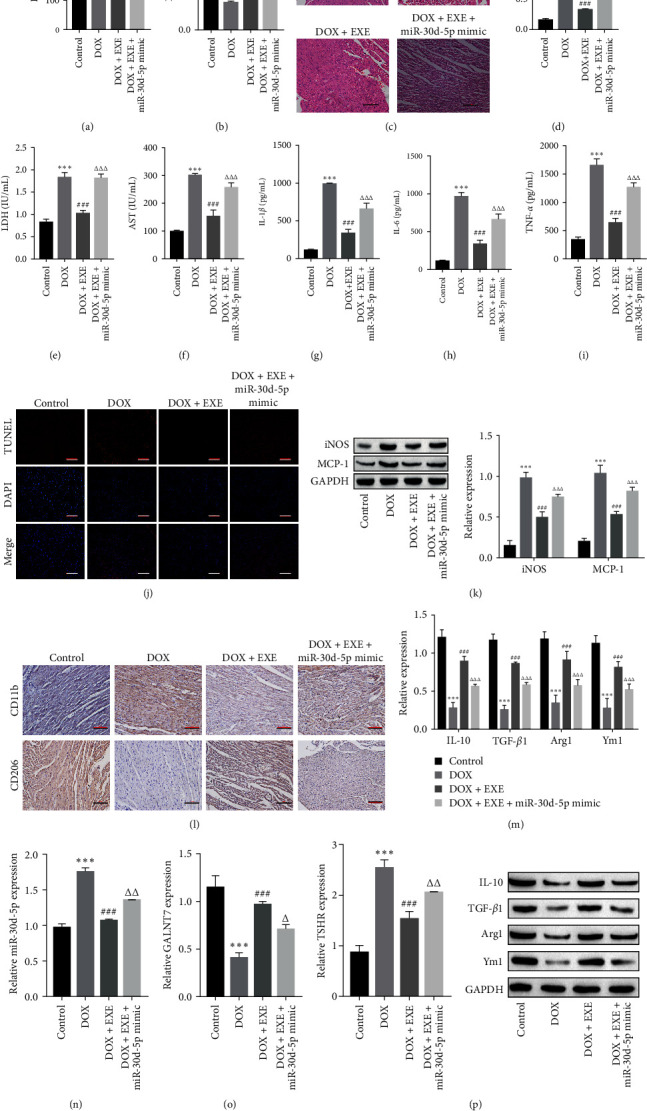
Exercise alleviates DOX-induced myocardial injury through miR-30d-5p. (A, B) Body and heart weights of experimental animals. (C) After transfection with the miR-30d-5p mimic, pathological changes in myocardial tissue were detected via H&E staining, scale bar = 100 μm. (D–F) The miR-30d-5p mimic increased the levels of myocardial injury indicators (cTnT, LDH, and AST) in rat serum. (G–I) ELISA revealed that the levels of the inflammatory factors IL-1*β*, IL-6, and TNF-*α* were increased after transfection with the miR-30d-5p mimic. (J) TUNEL analysis revealed that the miR-30d-5p mimic promoted cardiomyocyte apoptosis, scale bar = 100 μm. (K) Western blot analysis revealed that the miR-30d-5p mimic promoted the expression of the macrophage markers iNOS and MCP-1. (L) The expression of the macrophage markers CD11b and CD206 was detected by immunohistochemistry, scale bar = 100 μm. (M) Western blot analysis revealed that the miR-30d-5p mimic inhibited the expression of the macrophage markers IL-10, TGF-*β*1, Arg1, and Ym1. (N) RT-qPCR results revealed that the miR-30d-5p mimic promoted the expression of miR-30d-5p in rat myocardial tissue. (O) RT-qPCR results showed that the miR-30d-5p mimic inhibited the expression of GALNT7. (P) RT-qPCR results showed that the miR-30d-5p mimic promoted the expression of TSHR. One- or two-way ANOVA was used for statistical analysis. The data are expressed as mean ± SD. *⁣*^*∗∗∗*^*p* < 0.001 vs. Control; ^###^*p* < 0.001 vs. DOX; ^Δ^*p* < 0.05, ^ΔΔ^*p* < 0.01, ^ΔΔΔ^*p* < 0.001 vs. DOX + EXE. Control: normal rats without exercise intervention. AST, aspartate aminotransferase; cTnT, cardiac troponin T; DOX, doxorubicin; EXE, exercise; LDH, lactate dehydrogenase.

The results of the experimental animal body and heart weights revealed that the therapeutic effect of EXE intervention was attenuated after the overexpression of miR-30d-5p, and the body and heart weights of the rats were markedly lower than those of the DOX + EXE group (Figure 6A,B). H&E staining revealed that after overexpression of miR-30d-5p, the ability of EXE intervention to alleviate myocardial injury was attenuated, and the myocardial tissue structure was dispersed (Figure 6C). Compared with the DOX + EXE group, the overexpression of miR-30d-5p and EXE intervention after DOX treatment increased the expression levels of myocardial injury indicators and inflammatory factors (Figure 6D–I). The TUNEL results indicated that the apoptosis of cardiomyocytes increased after overexpressing miR-30d-5p (Figure 6J). The results of Western blot and immunohistochemistry revealed that, compared with that in the DOX + EXE group, macrophage polarization to the M2 phenotype was weakened after the overexpression of miR-30d-5p (Figure 6K‒M). The RT-qPCR data indicated that the levels of miR-30d-5p and TSHR were increased, whereas the level of GALNT7 was reduced after the overexpression of miR-30d-5p (Figure 6N‒P). In summary, EXE alleviates DOX-induced myocardial injury by downregulating the expression of miR-30d-5p, inhibiting inflammation and cardiomyocyte apoptosis, and inducing macrophage polarization to the M2 phenotype.

### 3.6. EXE Alleviates DOX-Induced Myocardial Injury Through miR-30d-5p/GALNT7

**Figure 7:**
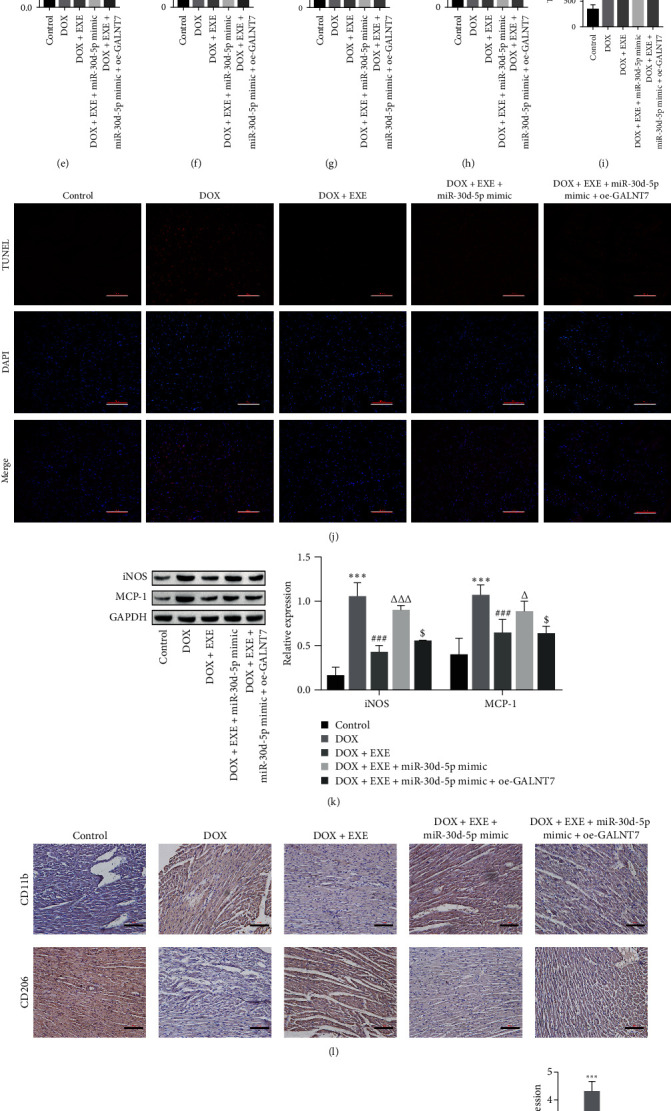
Exercise alleviates DOX-induced myocardial injury through miR-30d-5p/GALNT7. (A, B) Body weight and heart weight of experimental animals. (C) Pathological changes in myocardial tissue were observed via H&E staining, scale bar = 100 μm. (D–F) After overexpression of GALNT7, the serum myocardial injury indicators of the rats (cTnT, LDH, AST) decreased. (G–I) ELISA revealed that the levels of the inflammatory factors IL-1*β*, IL-6, and TNF-*α* decreased after overexpression of GALNT7. (J) TUNEL analysis revealed that oe-GALNT7 inhibited cardiomyocyte apoptosis, scale bar = 100 μm. (K) Western blot analysis revealed that oe-GALNT7 inhibited the expression of the macrophage markers iNOS and MCP-1. (L) The expression of the macrophage markers CD11b and CD206 was detected via immunohistochemistry, scale bar = 100 μm. (M) Overexpression of GALNT7 promoted the expression of the macrophage markers IL-10, TGF-*β*1, Arg1, and Ym1. (N) RT-qPCR revealed that oe-GALNT7 inhibited the expression of TSHR. One- or two-way ANOVA was used for statistical analysis. The data are expressed as the means ± SD. *⁣*^*∗∗∗*^*p* < 0.001 vs. Control; ^###^*p* < 0.001 vs. DOX; ^Δ^*p* < 0.05, ^ΔΔ^*p* < 0.01, ^ΔΔΔ^*p* < 0.001 vs. DOX + EXE; ^$^*p* < 0.05, ^$$^*p* < 0.01, ^$$$^*p* < 0.001 vs. DOX + EXE + miR-30d-5p mimic. Control: normal rats without exercise intervention. AST, aspartate aminotransferase; cTnT, cardiac troponin T; DOX, doxorubicin; EXE, exercise; LDH, lactate dehydrogenase; TSHR, thyroid stimulating hormone receptor.

The data of the experimental animal weights revealed that GALNT7 inhibited the effect of miR-30d-5p on EXE intervention and increased the body weight and heart weight compared with those of the DOX + EXE + miR-30d-5p mimic group (Figure 7A,B). H&E staining indicated that overexpression of GALNT7 attenuated the inhibitory effect of miR-30d-5p mimic on EXE intervention to alleviate DOX-induced myocardial injury compared with the DOX + EXE + miR-30d-5p mimic (Figure 7C). The overexpression of miR-30d-5p and GALNT7 together with EXE intervention after DOX treatment resulted in decreased expression levels of myocardial injury indicators and inflammatory factors (Figure 7D–I). TUNEL data revealed that overexpression of GALNT7 attenuated the effect of the miR-30d-5p mimic on EXE intervention and decreased apoptosis compared with that in the DOX + EXE + miR-30d-5p mimic group (Figure 7J). The results of Western blot and immunohistochemistry revealed that GALNT7 attenuated the effect of miR-30d-5p on EXE intervention, and the polarization of macrophages to M1 was reduced compared with that in the DOX + EXE + miR-30d-5p mimic group (Figure 7K–M). The RT-qPCR data revealed that, compared with that in the DOX + EXE + miR-30d-5p mimic group, the expression of TSHR was downregulated after GALNT7 was overexpressed (Figure 7N). In summary, EXE alleviates DOX-induced myocardial injury by downregulating the expression of miR-30d-5p, upregulating the expression of GALNT7, inhibiting inflammation and cardiomyocyte apoptosis, and inducing M2 polarization in macrophages.

### 3.7. EXE Attenuates DOX-Induced Myocardial Injury by Inhibiting TSHR Through GALNT7

**Figure 8:**
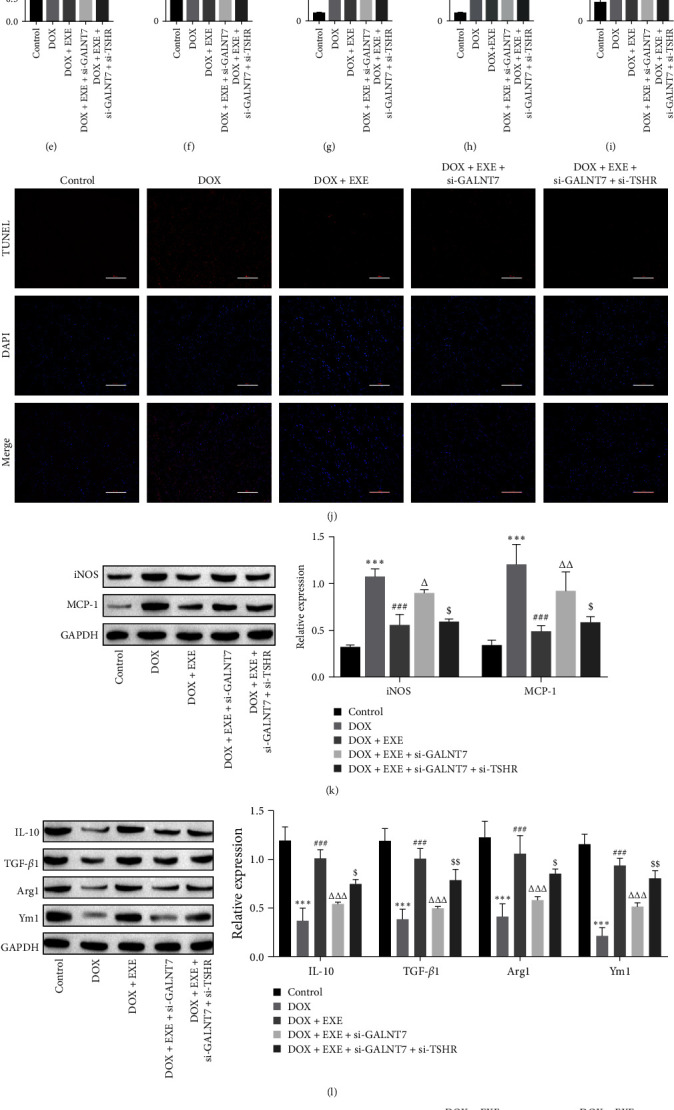
Exercise attenuates DOX-induced myocardial injury by inhibiting TSHR through GALNT7. (A, B) Body weight and heart weight of experimental animals. (C) Pathological changes in myocardial tissue were observed via H&E staining, scale bar = 100 μm. (D–F) si-GALNT7 increased the levels of serum myocardial damage indices (cTnT, LDH, and AST) in rats, and si-TSHR weakened the effect of si-GALNT7. (G–I) ELISA revealed that the levels of the inflammatory factors IL-1*β*, IL-6, and TNF-*α* were increased after transfection with si-GALNT7, and si-TSHR weakened the effect of si-GALNT7. (J) TUNEL indicated that apoptosis of cardiomyocytes increased after transfection with si-GALNT7 and decreased after further transfection with si-TSHR, scale bar = 100 μm. (K, L) The expression of the macrophage markers iNOS, MCP-1, IL-10, TGF-*β*1, Arg1, and Ym1 was detected by Western blotting. (M) The expression of the macrophage markers CD11b and CD206 was detected by immunohistochemistry, scale bar = 100 μm. One- or two-way ANOVA was used for statistical analysis. The data are expressed as mean ± SD. *⁣*^*∗∗∗*^*p* < 0.001 vs. control; ^###^*p* < 0.001 vs. DOX; ^Δ^*p* < 0.05, ^ΔΔ^*p* < 0.01, ^ΔΔΔ^*p* < 0.001 vs. DOX + EXE; ^$^*p* < 0.05, ^$$^*p* < 0.01, ^$$$^*p* < 0.001 vs. DOX + EXE + si-GALNT7. Control: normal rats without exercise intervention. AST, aspartate aminotransferase; cTnT, cardiac troponin T; DOX, doxorubicin; EXE, exercise; LDH, lactate dehydrogenase; SD, standard deviation; TSHR, thyroid stimulating hormone receptor.

The body weight and heart weight of the GALNT7 knockdown rats were decreased, whereas simultaneous knockdown of GALNT7 and TSHR attenuated the effects of GALNT7 on EXE intervention, and the body weight and heart weight increased (Figure 8A,B). H&E staining images revealed that GALNT7 knockdown attenuated the effect of EXE intervention, whereas simultaneous GALNT7 and TSHR knockdown attenuated the effect of si-GALNT7 on EXE intervention on DOX-induced myocardial injury (Figure 8C). Compared with the DOX + EXE group, the GALNT7-knockdown group presented increased expression levels of myocardial injury indicators and inflammatory factors, and simultaneous knockdown of GALNT7 and TSHR attenuated the effect of si-GALNT7 on EXE intervention and decreased the expression levels of myocardial injury indicators and inflammatory factors (Figure 8D–I). TUNEL data suggested that cardiomyocyte apoptosis increased after GALNT7 knockdown and decreased after further TSHR knockdown (Figure 8J). Western blot and immunohistochemistry results revealed that GALNT7 knockdown attenuated the effect of EXE on DOX-induced myocardial injury and that the macrophages were polarized to the M1 phenotype. After further knockdown of TSHR, the macrophages were polarized to the M2 phenotype (Figure 8K–M). In summary, EXE inhibits TSHR expression through GALNT7, inhibits inflammation and cardiomyocyte apoptosis, induces macrophage polarization to the M2 phenotype, and alleviates DOX-induced myocardial injury.

## 4. Discussion

DOX is an anthracycline cancer chemotherapeutic agent. EXE manifests as a physiological stress that triggers a beneficial adaptive cellular response that can induce a safeguard phenotype in the heart [30]. EXE is widely recognized as part of a healthy lifestyle and an effective strategy for nondrug treatment of cardiovascular disease. EXE can regulate a variety of signaling pathways and play an important role in myocardial protection [31]. However, the molecular mechanism by which EXE ameliorates DOX-induced myocardial injury still needs further study. In this study, we demonstrated that EXE alleviates DOX-induced myocardial injury through miR-30d-5p/GALNT7-mediated inhibition of TSHR-mediated regulation of macrophage polarization.

Earlier studies have shown that aerobic EXE can improve cardiac function and reverse the damage caused by chemotherapy drugs to the heart to some extent [32]. For example, EXE prevents ethanol-induced heart and liver damage in rats by inhibiting apoptosis and inflammation [33]. EXE intervention reduces acute and late DOX-induced cardiotoxicity [7, 8]. Consistent with earlier studies, our study revealed that DOX induces inflammation and promotes myocardial injury and apoptosis and that EXE intervention attenuates the effects of DOX on myocardial tissue. In addition, some studies have confirmed that EXE improves acute lung injury through the inhibition of the proinflammatory polarization of alveolar macrophages [34]. Other studies have shown that relatively high-intensity EXE increases macrophage M2 polarization, which can improve insulin resistance and inflammation caused by obesity [35]. Notably, in this study, we found that DOX-induced M1 polarization in macrophages, and EXE intervention induced M2 polarization in macrophages and inhibited M1 polarization, thus alleviating DOX-induced myocardial injury.

The abnormal expression of miR-30d-5p has been found to be associated with a variety of diseases [11]. Its mechanism of action is usually paired with 3′UTR complementary sequences on target mRNA transcripts, thus regulating the expression of target proteins and affecting the progression of diseases. For example, miR-30d-5p inhibits cell proliferation and autophagy [36]. miR-30d-5p inhibits the proliferation, migration, and invasion of lung squamous cell carcinoma by targeting DBF4 [37]. GALNT7 is a glycosyltransferase that can be targeted by multiple miRNAs to influence disease progression. For example, miR-125a-5p inhibits the proliferation and invasion of cervical cancer cells by inhibiting GALNT7 [38]. miR-363-3p regulates the expression of GALNT7 and promotes the occurrence and development of colorectal cancer [39]. In this study, we predicted a targeted binding site between miR-30d-5p and GALNT7 via StarBase and confirmed that miR-30d-5p targets GALNT7 by dual-luciferase reporter gene assay analysis. In addition, recent studies have shown that miR-30d-5p can target suppressor of cytokine signaling-1 (SOCS-1) and sirtuin 1 (SIRT1) to activate NF-kB signaling, thereby inducing macrophage M1 polarization and triggering macrophage apoptosis [14]. In this study, we found that overexpression of miR-30d-5p resulted in a decrease in GALNT7 expression and polarization of macrophages to M1, whereas overexpression of GALNT7 attenuated the effect of the miR-30d-5p mimic; that is, macrophages were polarized toward M2, the expression of the M1 macrophage markers CD11b and CD86 was significantly reduced, and the expression of the M2 macrophage markers CD206 and CD163 was significantly increased. Therefore, we demonstrated that miR-30d-5p negatively regulates GALNT7 targeting to inhibit M2 macrophage polarization. These findings suggest that the molecular axis of miR-30d-5 p/GALNT7 plays an important role in the regulation of macrophage polarization.

TSHR has been reported to promote macrophage inflammation by activating the mitogen-activated protein kinase (MAPK) and I*κ*B/p65 signaling pathways in macrophages [24]. In this study, we found that TSHR was enriched in the products of GALNT7 immune pretreatment by co-IP, and the expression of TSHR was significantly reduced after overexpression of GALNT7, indicating that TSHR and GALNT7 interact with each other and that GALNT7 negatively regulates TSHR expression. Moreover, our data revealed that overexpression of TSHR reversed the effect of GALNT7 overexpression, promoting the polarization of macrophages to the M1 phenotype and inhibiting the polarization of macrophages to the M2 phenotype. Therefore, GALNT7 regulates macrophage polarization by interacting with TSHR and downregulating the level of TSHR. In addition, after treating DOX-induced H9C2 cells with supernatant 3 (cocultured with the supernatant of THP-1 cells transfected with the miR-30d-5p mimic, oe-GALNT7, and oe-TSHR), cardiomyocyte apoptosis was promoted, and cell viability was inhibited. In this study, we reported for the first time that the miR-30d-5p/GALNT7/TSHR molecules interact with each other and regulate macrophage polarization, thereby affecting DOX-induced cardiomyocyte injury. These findings may provide a new therapeutic target for the treatment of myocardial cell injury.

Recent reports have indicated that miRNA expression changes under long-term EXE and that the expression level of miR-191a-5p in secreted extracellular vesicles in mice subjected to long-term EXE is downregulated [40]. Studies have shown that EXE training can regulate the expression of miRNAs in signaling pathways during cardiac fibrosis [41]. Bei et al. [42] reported that EXE can upregulate the expression of miR-486, thus alleviating myocardial ischemia‒reperfusion injury. In our study, after 6 weeks of EXE intervention in SD rats, the levels of miR-30d-5p and TSHR were significantly downregulated, the level of GALNT7 was increased, cardiomyocyte apoptosis was reduced, and the expression levels of myocardial injury indicators (cTnT, LDH, AST), IL-1*β*, IL-6, and TNF-*α* were reduced, M1 polarization was weakened, and M2 polarization was increased. These in vivo experimental results demonstrated that EXE regulates macrophage polarization through the molecular axis of miR-30d-5p/GALNT7/TSHR and alleviates myocardial injury. Previous research has shown that the same molecule or intervention may have different effects on different cell types [43]. We speculate that the miR-30d-5p/GALNT7/TSHR signaling pathway may also influence other cellular processes in myocardial injury. However, more experiments are needed. In addition, there are differences in the polarization of macrophages during different durations of EXE [44]. Therefore, future studies need to further explore whether the effect of EXE intervention on the polarization level of macrophages shows a progressive change over the 6-week period, which will help to understand the adaptation of the rat heart to EXE.

In this study, we investigated the molecular mechanism by which EXE regulates macrophage polarization through the miR-30d-5p/GALNT7/TSHR molecular axis at the cellular and animal levels and subsequently alleviates DOX-induced myocardial injury. However, this study has several limitations. First, our study is based on a rat animal model, and it remains to be seen whether the same conclusions would be drawn in other species. In addition, different EXE intensities and durations are needed to fully understand the therapeutic effects. Second, we lack clinical trials to verify whether the conclusions of these animal experiments are clinically valid and feasible. In future studies, we will further investigate the long-term effects of EXE on miR-30d-5p/GALNT7/TSHR expression in clinical settings and explore other miRNAs that may be involved in EXE-induced cardiac protection.

## 5. Conclusion

In summary, our research group found that EXE regulates macrophage polarization through the miR-30d-5p/GALNT7/TSHR axis and subsequently alleviates DOX-induced myocardial injury. These results show that EXE could be a viable nonpharmacological intervention to mitigate DOX-induced cardiotoxicity and that targeting the miR-30d-5p/GALNT7/TSHR axis might offer new therapeutic strategies for preventing myocardial injury.

## Figures and Tables

**Table 1:** RT-qPCR primer sequences.

Target gene	The sequence of primers: 5′-3′
miR-30d-5p	F: 5′-TGTAAACATCCCCGACTGGA-3′
	R: 5′-GCGAGCACAGAATTAATACGAC-3′

GALNT7	F: 5′-CCAAGAAGAATGCAAGTATTGG-3′
	R: 5′-CATCCTTCATTATGGAAGACGA-3′

TSHR	F: 5′-ACAGACAACCCTTACATGAC-3′
	R: 5′-GTATAGTTTCAGGGTCAAGGT-3′

GAPDH	F: 5′-AACTCCCATTCTTCCACCT-3′
	R: 5′-TTGTCATACCAGGAAATGAGC-3′

U6	F: 5′-CTCGCTTCGGCAGCACA-3′
	R: 5′-AACGCTTCACGAATTTGCGT-3′

Abbreviation: RT-qPCR, reverse transcription-quantitative polymerase chain reaction.

## Data Availability

The datasets used and/or analyzed during the current study are available from the corresponding author upon reasonable request.
